# The Role of Node Heterogeneity in the Coupled Spreading of Epidemics and Awareness

**DOI:** 10.1371/journal.pone.0161037

**Published:** 2016-08-12

**Authors:** Quantong Guo, Yanjun Lei, Chengyi Xia, Lu Guo, Xin Jiang, Zhiming Zheng

**Affiliations:** 1 School of Mathematics and Systems Science, Beihang University & Key Laboratory of Mathematics Informatics Behavioral Semantics(LMIB), Beijing 100191, China; 2 School of Mathematical Sciences, Peking University, Beijing 100191, China; 3 Tianjin Key Laboratory of Intelligence Computing and Novel Software Technology, Tianjin University of Technology, Tianjin 300384, China; 4 Key Laboratory of Computer Vision and System (Ministry of Education),Tianjin University of Technology, Tianjin 300384, China; 5 Luoyang Branch of China Construction Bank, Luoyang 471000, China; Wenzhou University, CHINA

## Abstract

Exploring the interplay between information spreading and epidemic spreading is a topic that has been receiving increasing attention. As an efficient means of depicting the spreading of information, which manifests as a cascade phenomenon, awareness cascading is utilized to investigate this coupled transmission. Because in reality, different individuals facing the same epidemic will exhibit distinct behaviors according to their own experiences and attributes, it is important for us to consider the heterogeneity of individuals. Consequently, we propose a heterogeneous spreading model. To describe the heterogeneity, two of the most important but radically different methods for this purpose, the degree and k-core measures, are studied in this paper through three models based on different assumptions. Adopting a Markov chain approach, we succeed in predicting the epidemic threshold trend. Furthermore, we find that when the k-core measure is used to classify individuals, the spreading process is robust to these models, meaning that regardless of the model used, the spreading process is nearly identical at the macroscopic level. In addition, the k-core measure leads to a much larger final epidemic size than the degree measure. These results are cross-checked through numerous simulations, not only of a synthetic network but also of a real multiplex network. The presented findings provide a better understanding of k-core individuals and reveal the importance of considering network structure when investigating various dynamic processes.

## Introduction

As one of the most important dynamic processes in complex networks, the diffusion process [1, 2], especially the spreading of epidemics [3–9], has been receiving increasing interest for decades. Various models have been developed to describe the diffusion of epidemics [10], rumors [11], innovation [12] and so on. Different factors [13–15], such as the frequency of contact between individuals, the disease duration, and the immunity of particular individuals, have been considered in these models to provide a realistic and comprehensive understanding of the spreading process. In particular, as an important representation of the spread of information about epidemics, awareness spreading [16–20] has attracted increasing interest. A pioneering step in this direction was taken by Funk et al. [16], who studied the spreading of epidemics while accounting for the spread of awareness. Their results show that the final epidemic size can be markedly reduced, whereas the epidemic threshold can be reduced only when the awareness is sufficiently strong. At the same time, due to the different spreading paths of awareness and epidemics, considering the spreading of the coupled dynamic process only on the same single-layer network makes it difficult to obtain a comprehensive understanding of the process as a whole. As a result, as a natural means of describing mixed complex systems, methods based on multiplex networks [21–30] have recently found success in revealing abundant features of various dynamic processes, including epidemic spreading. Within the framework of multiplex networks, Clara *et al.* [31, 32] developed a UAU-SIS model and found that a metacritical point exists from which an epidemic can be delayed and contained. Guo *et al.* [33] proposed a local awareness-controlled contagion spreading model in which the awareness layer is a threshold model and also used the same framework as a UAU-SIS model to analyze the problem. The analytical results showed high accuracy compared with Monte Carlo simulations.

**Fig 1:**
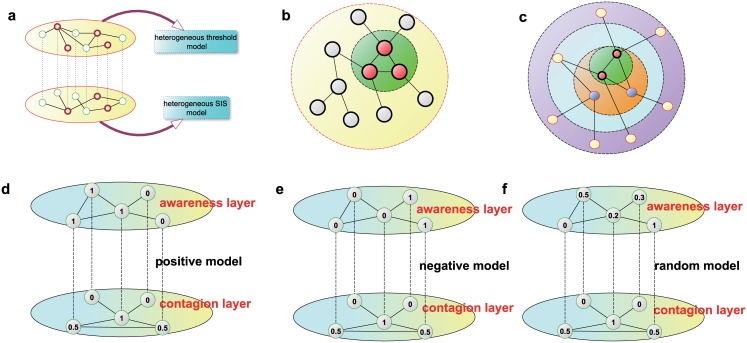
Illustration of the heterogeneous LACS model. (a) A schematic illustration of a two-layer multiplex network in which the upper layer represents the spreading of awareness and the lower layer is the contagion layer. The spreading models and topological structures of these two layers are different from each other. A threshold model is used on the awareness layer, whereas an SIS model is used on the contagion layer. Red nodes represent individuals that are aware or infected. (b-c) Diagrammatic representations of a simple network under the k-core decomposition and the degree decomposition, respectively. It is clear that the degree decomposition is considerably different from the k-core decomposition. The two nodes of degree k = 3 in the network are in different locations: one is in the innermost core of the network, whereas the other lies at the periphery. (d-f) Toy models of the linear positive correlation model, the linear negative correlation model and the random model, respectively, for the local awareness threshold *α*. The values on the awareness layer correspond to the local awareness threshold *α*, whereas the values on the contagion layer correspond to the reduction rate *γ*. Here, we use the k-core index as the measure of the heterogeneity of individuals on the awareness layer, whereas the degree is the measure used on the contagion layer.

However, in these studies, there exists a hidden assumption that all individuals are treated equally. In fact, because of the complex topological structures of networks, the discrepancies among different individuals have a significant influence on the epidemic spreading process [34], as in other spreading processes [35, 36]. The large heterogeneity of the resulting network strongly determines the efficiency and speed of spreading [37–41]. For instance, if a node is a hub with a large number of neighbors, then even if the corresponding individual is aware of the epidemics, it is still relatively easy for that individual to become infected; therefore, the reductive effect of awareness on the infection rate is not as strong for these individuals. Consequently, it is essential to consider the heterogeneity of different individuals in the epidemic spreading process. Moreover, because the spreading of awareness, which can always be modeled as a threshold model [42], has a significant influence on epidemic spreading, there is also a need to introduce a heterogeneous threshold model instead of a homogeneous threshold model [33]. Therefore, as shown in Fig 1(a), the purpose of this paper is to develop a heterogeneous spreading model, on both the awareness layer and the contagion layer, to integrate these two issues so as to gain a deeper understanding of the interplay between the spreading of awareness and epidemics.

Here, to account for the heterogeneity of individuals, we must first introduce the measures that can be used to classify individuals into different groups. A number of different measures aimed at ranking the importance of individuals have been suggested in recent years [43–45], of which the most direct and widely used topological measure is the node degree [3, 36]. In a complex network with a broad degree distribution, the hubs (individuals with the most connections) are usually believed to be responsible for the largest spreading processes [3, 36, 45]. Furthermore, as a global measure (in contrast to the local degree measure), k-core (also called k-shell) decomposition analysis has recently revealed abundant details concerning the importance of individuals to spreading processes in social networks [45–47]. The k-core measure is used to describe the location of an individual by assigning an integer index *k*_*s*_ and pruning all individuals with *k* ≤ *k*_*s*_. Peripheral individuals have small *k*_*s*_ values, whereas a large *k*_*s*_ corresponds to the core of the network [46]. This measure shows that there are plausible circumstances in which the true “hubs” of the network do not correspond to the most highly connected nodes; instead, the individuals located within the core of the network may be the true “hubs”. Schematic representations of a network in terms of the degree and k-core measures are shown in Fig 1(b) and 1(c).

In this paper, for simplicity and completeness, we use the degree and k-core indexes as measures of individual heterogeneity to explore its effects on epidemic spreading. With the heterogeneity taken into account, we find that in epidemic spreading, the k-core measure is robust to the awareness heterogeneity of the nodes, meaning that the epidemic spreading process remains nearly identical under different awareness heterogeneity models. In addition, the heterogeneity of individuals as indicated by the k-core measure can also promote the spreading of epidemics, with an especially strong influence on the final epidemic size. The results are established through analytical methods based on extensive numerical simulations of correlated and uncorrelated multiplex networks. Moreover, as an application example, we apply our model to the human HIV1 multiplex network [48, 49] and obtain the same findings, thus providing a better understanding of the effects of the heterogeneity of individuals on the spreading of epidemics. Last but not least, the results also provide a route toward an efficient strategy for suppressing the spread of epidemics.

## Results

To clearly present our results, we first describe our two-layer multiplex network model of epidemic spreading considering the cascading of awareness. For simplicity, we assume that the multiplex network is unweighted and undirected.

### The local awareness controlled contagion spreading model on a multiplex network

The local awareness controlled contagion spreading (LACS) model consists of two layers, of which the upper layer represents the awareness states of the individuals and the lower layer corresponds to the physical epidemic states of the individuals. Each individual in the awareness layer has a one-to-one correspondence with its counterpart on the contagion layer. The structures of the two layers can, in general, be quite different, because the upper layer represents the spreading of information, whereas the spreading of the epidemic occurs on the lower layer. On the awareness layer, if an individual is aware of the epidemic, it is in the aware (A) state; otherwise, it is in the unaware (U) state. Correspondingly, on the contagion layer, if an individual is infected, it is in the infected (I) state; otherwise, its state is susceptible (S). Individuals on the awareness layer spread awareness of the epidemic while the contagion process proceeds on the contagion layer; the awareness layer is a threshold model, and the contagion layer is a classical SIS model. Specifically, the evolution of the dynamic awareness process is defined as follows. On the one hand, two cases exist in which an unaware individual can become aware: (1) the individual becomes infected, or (2) the ratio between the number of its neighbors that are aware and its degree (the number of links connected to it) surpasses the critical point, which we call the local awareness threshold *α*. On the other hand, an aware individual can revert to the unaware state with a probability *δ*. Additionally, on the contagion layer, a susceptible individual can be infected by an infectious neighbor with probability *β*, whereas an infected individual can recover to the susceptible state with probability *μ*. We use *β*^*A*^ or *β*^*U*^ to denote the infection rate when an individual is aware or unaware, respectively. These rates are different because if an individual is aware of the epidemic, it may take measures to protect itself, thereby leading to a reduced infectivity, *β*^*A*^ = *γβ*^*U*^. Each individual can only be in one of three states: unaware and susceptible (US), aware and infected (AI), or aware and susceptible (AS).

### The heterogeneous LACS model on a multiplex network

As defined above, to account for the heterogeneity of individuals on both the contagion layer and the awareness layer, we should investigate the details of the coupled dynamic process. The two diffusion processes are coupled through the reduction of the infection rate *β* on the contagion layer if an individual is aware on the awareness layer. However, the reduction rate 1 − *γ* should differ among different individuals because of their heterogeneity, which means that the contagion layer should be a heterogeneous SIS model. In reality, it is more difficult for a more important individual to remain unaffected by an epidemic, which suggests that the reduction rate 1 − *γ* for such individuals should be smaller. This is easy to understand based on the fact that the entire system depends predominantly on certain key individuals. If an individual is a key player, then in some sense, the connections with that individual are more important than those with others. Therefore, the characteristic of high activity leads to a smaller reduction 1 − *γ* in the infection rate. Here, we propose a linear model, one of the simplest possible approaches, to assign each individual a value of *γ* according to that individual’s importance. Because we use the degree and k-core measures in this paper to quantify the importance of individuals, the linear model of the reduction rate is defined as follows:

$$\gamma_{i}=\frac{k_{i}-k_{min}}{k_{max}-k_{min}}\;\text{or}\;\gamma_{i}=\frac{k_{s}^{i}-k_{s}^{min}}{k_{s}^{max}-k_{s}^{min}}$$

where *k*_*max*_ and *k*_*min*_ represent the maximum and minimum degrees, respectively, of individuals on the contagion layer. Equivalently, $k_{s}^{max}$ and $k_{s}^{min}$ are the maximum and minimum k-core indexes, respectively, of individuals on the contagion layer. The degree and k-core indexes of an individual *i* are *k*_*i*_ and $k_{s}^{i}$, respectively.

On the awareness layer, to account for the heterogeneity of individuals, different values of the local awareness threshold *α* are assigned to each individual. For completeness and simplicity, we propose three models, including a random model, a linear positive correlation and a linear negative correlation, for the assignment of a local awareness threshold *α*_*i*_ to individual *i*:

1. Linear positive correlation model
  This model assumes that the local awareness threshold *α* and the degree or k-core index of an individual on the awareness layer have a linear positive correlation. This assumption implies that an individual with a larger degree or k-core index has a larger local awareness threshold *α*, which makes it more difficult for the individual to become aware:
  $$\alpha_{i}=\frac{k_{i}-k_{min}}{k_{max}-k_{min}}\;\text{or}\;\alpha_{i}=\frac{k_{s}^{i}-k_{s}^{min}}{k_{s}^{max}-k_{s}^{min}}$$
  where *k*_*max*_, *k*_*min*_, $k_{s}^{max}$, $k_{s}^{min}$, *k*_*i*_, and $k_{s}^{i}$ have the same meanings as those on the contagion layer but are based on the topological structure of the awareness layer.
2. Linear negative correlation model
  In contrast with the linear positive correlation model, this model assumes that on the awareness layer, the local awareness threshold *α* and the degree or k-core index of an individual have a linear negative correlation, which means that an individual with a larger degree or k-core index has a smaller local awareness threshold *α*. In other words, an individual can more easily become aware if its degree or k-core index is large:
  $$\alpha_{i}=\frac{k_{max}-k_{i}}{k_{max}-k_{min}}\;\text{or}\;\alpha_{i}=\frac{k_{s}^{max}-k_{s}^{i}}{k_{s}^{max}-k_{s}^{min}}$$
  where *k*_*max*_, *k*_*min*_, $k_{s}^{max}$, $k_{s}^{min}$, *k*_*i*_, and $k_{s}^{i}$ also have the same meanings as for the linear positive correlation model.
3. Random model
  In the random model, we randomly assign different values of *α* in the range [0, 1] to different individuals on the awareness layer. Note that individuals with the same degree or k-core index may have different local awareness thresholds:
  $$\alpha_{i}=random\;\left[0,1\right]$$

We use a toy model to illustrate these three models on the awareness layer in Fig 1(d), 1(e) and 1(f). Because the reduction rate is simply related to the topological structure of the contagion layer, it is clear that regardless of the model used on the awareness layer, the reduction rate for individuals on the contagion layer remains the same.

### The coupled dynamic process in the heterogeneous LACS model

For describing an epidemic, the epidemic threshold and the final epidemic size are two key quantities. Hence, we develop a simple method of calculating epidemic thresholds using different heterogeneity models. In particular, we adopt the MMCA method [31] (see the Methods section for details) to analyze these models. So as to help readers have a clear understanding of our model, in Table 1, we list the meanings of some key parameters used in our model.

**Table 1:** The details of some key parameters.

parameter	description
*α*_*i*_	local awareness threshold for node i
*δ*	transition probability from aware to unaware
*β*^*U*^	infection rate for unaware individual
*β*^*A*^	infection rate for aware individual
*μ*	recovery rate for infected individual
*γ*_*i*_	reduced infection rate of aware node i
*k*_*i*_	degree of node i
$k_{s}^{i}$	k-core index of node i
*P*^*I*^(*s*)	average infection probability of nodes with degree s or k-core index
*P*^*A*^(*s*)	average awareness probability of nodes with degree s or k-core index

According to the heterogeneous LACS model defined above, let *A* = {*a*_*ij*_}_*N* × *N*_ and *B* = {*b*_*ij*_}_*N* × *N*_ be the adjacency matrices of the awareness layer and the contagion layer, respectively, where *N* is the number of individuals on each layer. Then, the probability that individual *i* will not change from state *U* to state *A* is *r*_*i*_, and the probability that it will not be infected by any neighbor is $q_{i}^{A}$, if it is aware, or $q_{i}^{U}$, if it is unaware. For these probabilities with respect to time, we have

$$r_{i}\left(t\right)=H\left(\alpha_{i}-\frac{\sum_{j}a_{ji}p_{j}^{A}\left(t\right)}{k_{i}}\right)$$ (1)

$$q_{i}^{A}\left(t\right)=\Pi \left(1-b_{ji}p_{j}^{AI}\left(t\right)\beta_{i}^{A}\right)$$ (2)

$$q_{i}^{U}\left(t\right)=\Pi \left(1-b_{ji}p_{j}^{AI}\left(t\right)\beta^{U}\right)$$ (3)

where $p_{j}^{A}\left(t\right)$ and $p_{j}^{AI}\left(t\right)$ represent the probabilities that individual *j* is aware or infected, respectively, at time *t*. In addition, **H**(*x*) is a Heaviside step function, i.e., if *x* > 0, then **H**(*x*) = 1, and otherwise, **H**(*x*) = 0. In other words, *r*_*i*_(*t*) can only take a value of 1 or 0, depending on the awareness states of its neighbors. These equations are obtained by supposing that the contributions from all neighbors are independent of each other, which is the only approximation adopted in the MMCA method [31, 32].

As a result of the MMCA method, we obtain the epidemic threshold $\beta_{c}^{U}$, which is

$$\beta_{c}^{U}=\frac{\mu }{\Lambda_{max}}$$

The calculation of the epidemic threshold $\beta_{c}^{U}$ reduces to the solution of an eigenvalue problem. Specifically, let *S* be the matrix whose elements are $s_{ji}=[1-\left(1-\gamma_{i}\right)p_{i}^{A}]b_{ji}$; then, Λ_*max*_ is the maximal eigenvalue of *S*.

### Simulations on different synthetic networks

To examine the effects of the heterogeneity of individuals on the coupled dynamic process, simulations performed on different synthetic networks are presented below. Because our model consists of two layers and each layer has its own measure of individual heterogeneity, either the degree or the k-core measure, four cases of the heterogeneity measures used in the multiplex network exist: (1) the degree measure versus the degree measure, (2) the degree measure versus the k-core measure, (3) the k-core measure versus the degree measure, and (4) the k-core measure versus the k-core measure. Recent works have shown that interlayer correlations can have a significant impact on the dynamic process in a multiplex network [50–52]. In reality, a positive interlayer correlation is more common than a negative correlation [22, 53]; for example, an individual who is important or has many links in one type of social network tends to also have many links in other types of networks. Therefore, in this paper, we study only positively correlated and uncorrelated multiplex networks.

#### Dynamic spreading process on a positively correlated multiplex network

**Fig 2:**
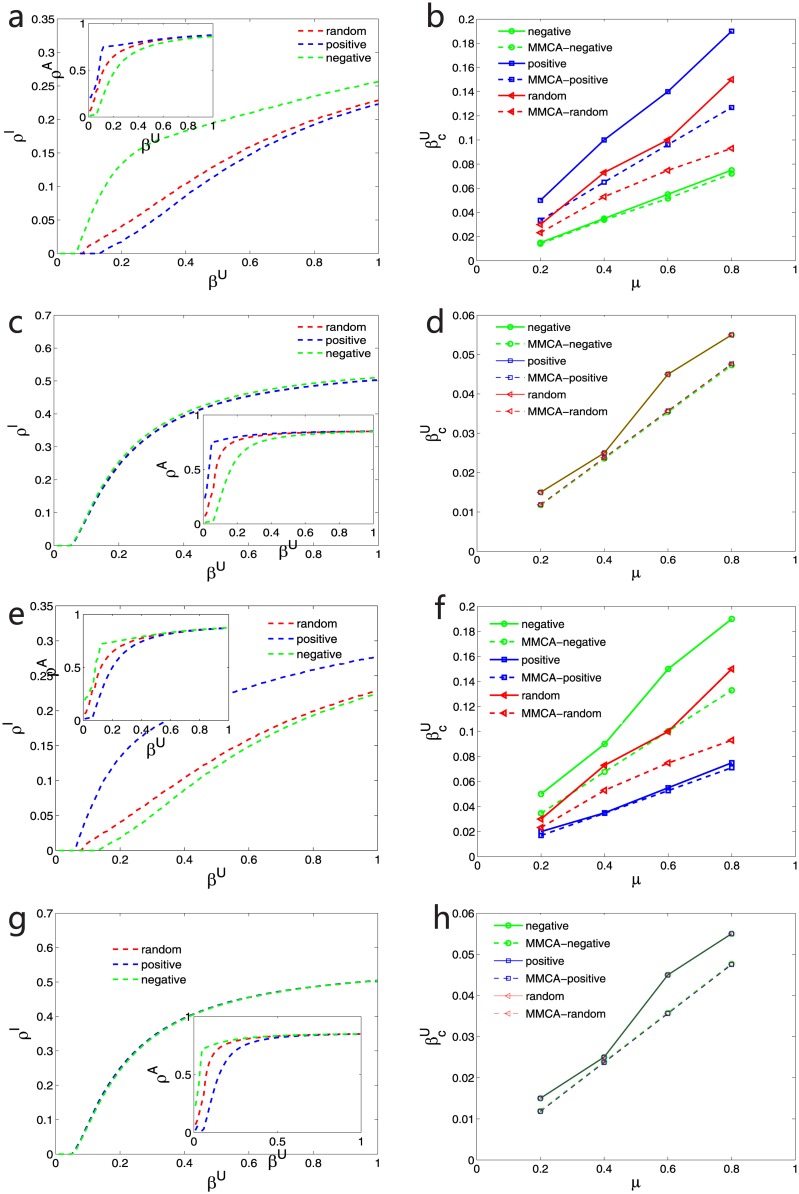
For SF-SF networks, the effects of the three heterogeneity models on the coupled dynamic process and epidemic threshold calculations in the MMCA method. The four cases of the heterogeneity measures used in the multiplex network are as follows: (a) and (b) degree measure versus degree measure, (c) and (d) degree measure versus k-core measure, (e) and (f) k-core measure versus degree measure, and (g) and (h) k-core measure versus k-core measure. In (a), (c), (e), and (g), each panel shows the stationary fractions of infected individuals (*ρ*^*I*^) and aware individuals (*ρ*^*A*^; the insets) obtained from Monte Carlo simulations as a function of the infectivity *β* under the random model (red dotted line), the linear positive correlation model (blue dotted line) and the linear negative correlation model (green dotted line). The other parameters are set as follows: *μ* = 0.8 and *δ* = 0.3. In (b), (d), (f), and (h), comparisons of the epidemic thresholds between the Monte Carlo simulations (solid line) and the MMCA method (dotted line) are presented. Note that *δ* = 0.1 for all curves in these four panels.

Fig 2 shows the results obtained on an SF multiplex network. The two layers have the same SF network structure with 10^4^ nodes, which was generated using the configuration model [54] with an exponent equal to 3. Because the topological structure of both layers is the same, the multiplex network has the maximal positive correlation [50, 51], which is the simplest case for a correlated multiplex network. Meanwhile, the average degree 〈*k*〉 of the network is 6. To initiate an epidemic spreading process, 20% of the nodes on the contagion layer were set as infected, with their counterparts on the awareness layer consequently aware of the epidemic. In the heterogeneous LACS model, each node on the contagion layer has a fixed reduction rate *γ* according to its degree or k-core measure on the contagion layer, whereas on the awareness layer, each node has a different value of the local awareness threshold *α* under different heterogeneity models. The rules governing the coupled dynamic process were iterated with parallel updating until the total percentage of infected nodes *ρ*^*I*^ reached a steady state. As seen from Fig 2(a), 2(c), 2(e) and 2(g), the spreading process strongly depends on the heterogeneity measure on the contagion layer. In particular, when the k-core measure is used on the contagion layer, the measure on the awareness layer and the heterogeneity model have little effect on the epidemic spreading behavior. However, when the heterogeneity measure used on the contagion layer is the degree measure, the use of different measures on the awareness layer results in significantly different spreading process for the same models, in terms of not only the epidemic threshold but also the final epidemic size. For instance, in Fig 2(a), the degree measure is used on the awareness layer and the spreading process in the negative model is more rapid than in the other models, which means that the epidemic threshold is smaller and the final epidemic size is larger; meanwhile, the opposite occurs when the k-core measure is used on the awareness layer, as seen from Fig 2(e). These phenomena indicate that the spreading process is ‘robust’ when the infection rate of each node is assigned according to the k-core measure on the contagion layer, regardless of the heterogeneity model used on the awareness layer. In other words, as a representation of information spreading, because the awareness heterogeneity has little effect on the epidemic spreading behavior when we classify the contagion characteristics of the nodes according to their k-core indexes, the k-core index is an epidemic spreading structure that is ‘robust’ to the cascading of awareness. Moreover, it is also clear that when the k-core measure is used on the contagion layer, the final epidemic size is significantly larger than that obtained for the degree measure. In our model, according to the definition of the reduction rate *γ*, regardless of whether the degree measure or the k-core measure is used, a more important node can more easily become infected. Therefore, the larger final epidemic size obtained when the k-core measure is used on the contagion layer reveals that this measure, in some sense, reflects the role of a node as an influential spreader [45].

Moreover, to gain a better understanding of the effects induced by the coupled dynamic process on the awareness layer, it is also interesting for us to investigate the spreading of awareness. In the insets of Fig 2(a), 2(c), 2(e) and 2(g), we observe that the spreading of awareness also relies primarily on the awareness layer. It is clear that the stationary fraction of aware nodes, *ρ*^*A*^, exhibits two different spreading processes with increasing *β* regardless of the heterogeneity model used on the awareness layer. In the first spreading process, when *β* is small, different models appear to have different *ρ*^*A*^ values, but the differences become smaller as *β* increases. This suggests the necessity of examining the coupled dynamic process at different *β* values. Additionally, we also compare the MMCA method with Monte Carlo simulations for calculating the epidemic thresholds, as shown in Fig 2(b), 2(d), 2(f) and 2(h). When the k-core measure is used on the contagion layer, the MMCA method exhibits good accuracy; however, when the degree measure is used on the contagion layer, the epidemic thresholds obtained using the MMCA method are smaller than those obtained in the Monte Carlo simulations, although the discrepancy is smaller for small epidemic thresholds. For example, in Fig 2(b), the epidemic threshold for the positive model is larger than that for the negative model, and the MMCA method does not agree as well with the Monte Carlo simulations for the negative model. The reason is as follows: since we assume that the contributions from all neighbors are independent in the MMCA method [31], in our heterogeneous LACS model when the heterogeneity measure of the contagion layer is degree, the value of *r*_*i*_, the probability that an unaware individual *i* will remain unaware, is smaller than that in the Monte Carlo simulations in many cases; thus, it is much easier for an outbreak to occur. This phenomenon also indicates that in the coupled spreading process, compared with degree measure, k-core measure is a better way to classify the heterogeneity of nodes.

**Fig 3:**
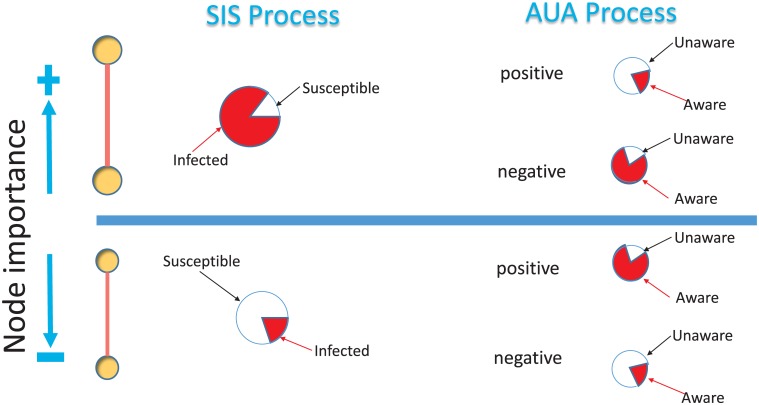
Illustration of the probabilities of becoming infected and aware for two groups of nodes under different models. A larger sectorial area corresponds to a greater probability. For the upper group of nodes, which are of greater importance, the probability of infection is larger. Meanwhile, the awareness probability is smaller under the positive model and larger under the negative model. The opposite situation arises for the lower group of nodes: the infection probability and awareness probability are smaller under the negative model, whereas under the positive model, the awareness probability is larger.

To explain why the different models yield remarkably diverse results, we classify the nodes into two groups for simplicity. The first group contains nodes with larger degree or k-core indexes, whereas the other includes nodes with smaller degree or k-core indexes, as seen in Fig 3. Because the multiplex network is positively correlated, a ‘hub’ on one layer is also a ‘hub’ on the other layer. Therefore, in the following, by considering the occasions when the measures on the two layers are the same, we qualitatively analyze the coupled dynamic process. When the degree measure is used on both layers and the positive model is used on the awareness layer, nodes with larger degrees tend to more easily become infected and enter the IA state, whereas it is easier for nodes with smaller degrees to be in the SA state. However, if the negative model is used on the awareness layer, nodes with smaller degrees are more likely to be in the SU state. Because awareness can reduce infectivity, epidemic outbreaks can more easily occur in the negative model. In the case of the k-core measure, according to the calculation of the k-core index, nodes with larger indexes may not have as many links because the distribution of the k-core index is not as broad as the degree distribution [46]. Therefore, unlike the case of the degree measure, it is easier for some nodes with larger k-core indexes to be in the SU state when the positive model is used on the awareness layer. This factor accelerates epidemic spreading, resulting in a much smaller discrepancy between the positive model and the negative model than in the case of the degree measure. Thus, the robustness of the k-core measure to different models arises because of the balance of these factors.

**Fig 4:**
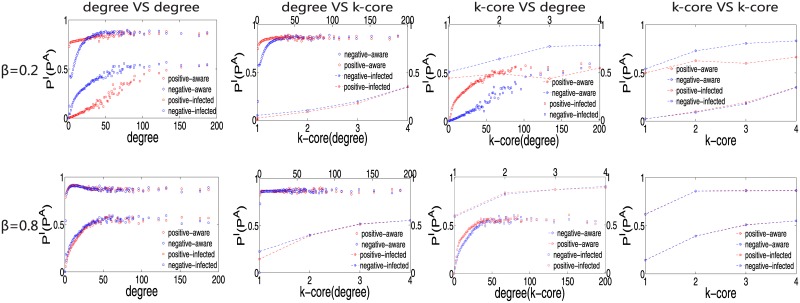
The average infection (awareness) probability *P*^*I*^ (*P*^*A*^) as a function of the degree or k-core index. The results for the four cases of different combinations of heterogeneity measures are shown for both the positive and negative models: (1) degree versus degree, (2) degree versus k-core, (3) k-core versus degree, and (4) k-core versus k-core. For each case, we compare the results for two *β* values, 0.2 and 0.8. The other parameters are set as follows: *μ* = 0.8 and *δ* = 0.3. The x axis represents the degree or k-core index, and the y axis represents the probability of being infected or aware. All simulations were performed on the same SF multiplex network defined above.

Because the process of awareness spreading is distinct from that of epidemic spreading, to explore the details of these spreading processes, we consider the average infection (awareness) probability *P*^*I*^ (*P*^*A*^) of nodes with a given degree or k-core index. The infection (awareness) probability is averaged over all nodes with the same degree or k-core index as follows:

$$P^{I}\left(k\right)\left(P^{A}\left(k\right)\right)=\sum_{i=k}\frac{P^{I}\left(i\right)}{M(k)}\left(\sum_{i=k}\frac{P^{A}\left(i\right)}{M(k)}\right)$$

where *P*^*I*^(*i*) (*P*^*A*^(*i*)) is the probability that node *i* is infected (aware) in the epidemic (awareness) spreading process and *M*(*k*) represents the number of nodes for which the degree or k-core index is equal to k. In Fig 4, we illustrate the analysis of *P*^*I*^(*k*) (*P*^*A*^(*k*)) in the four cases of different combinations of heterogeneity measures on the same multiplex network defined above. Considering that different values of *β* have obvious effects on the stationary fraction of aware nodes, we investigate two scenarios: *β* = 0.2 and *β* = 0.8. Many interesting findings are revealed in the figure, not only with respect to epidemic spreading but also with respect to awareness spreading. Let us first consider the scenario of *β* = 0.2. We find that when the degree measure is used on the contagion layer, the infection probability *P*^*I*^(*k*) has a wide range, which means that *P*^*I*^(*k*) is not a monotonic function of the degree. However, when the k-core measure is used on the contagion layer, *P*^*I*^(*k*) increases monotonically as the k-core index increases. Moreover, on the awareness layer, the opposite situation is observed. The results show that for the k-core measure, *P*^*A*^(*k*) is not a monotonic function, whereas it is very similar to one for the degree measure. Therefore, this indicates that because of the coupled dynamic process, when *β* is small, hubs are not always ‘hubs’ in the spreading of epidemics and nodes with larger k-core indexes are also not always ‘hubs’ in awareness spreading. By contrast, the k-core measure exhibits a stable monotonicity on both layers when *β* is large, for instance, *β* = 0.8. These phenomena suggest that for the contagion layer, the k-core measure is a suitable heterogeneity measure for classifying nodes into different groups in the heterogeneous model. At the same time, Fig 4 also helps us understand the differences between *ρ*^*I*^ and *ρ*^*A*^ in different models. For example, the spreading of epidemics is robust to the heterogeneity model when the k-core measure is used on the contagion layer because in this case, the infection probability *P*^*I*^ is nearly identical for every k-core index.

#### Dynamic spreading process on an uncorrelated multiplex network

**Fig 5:**
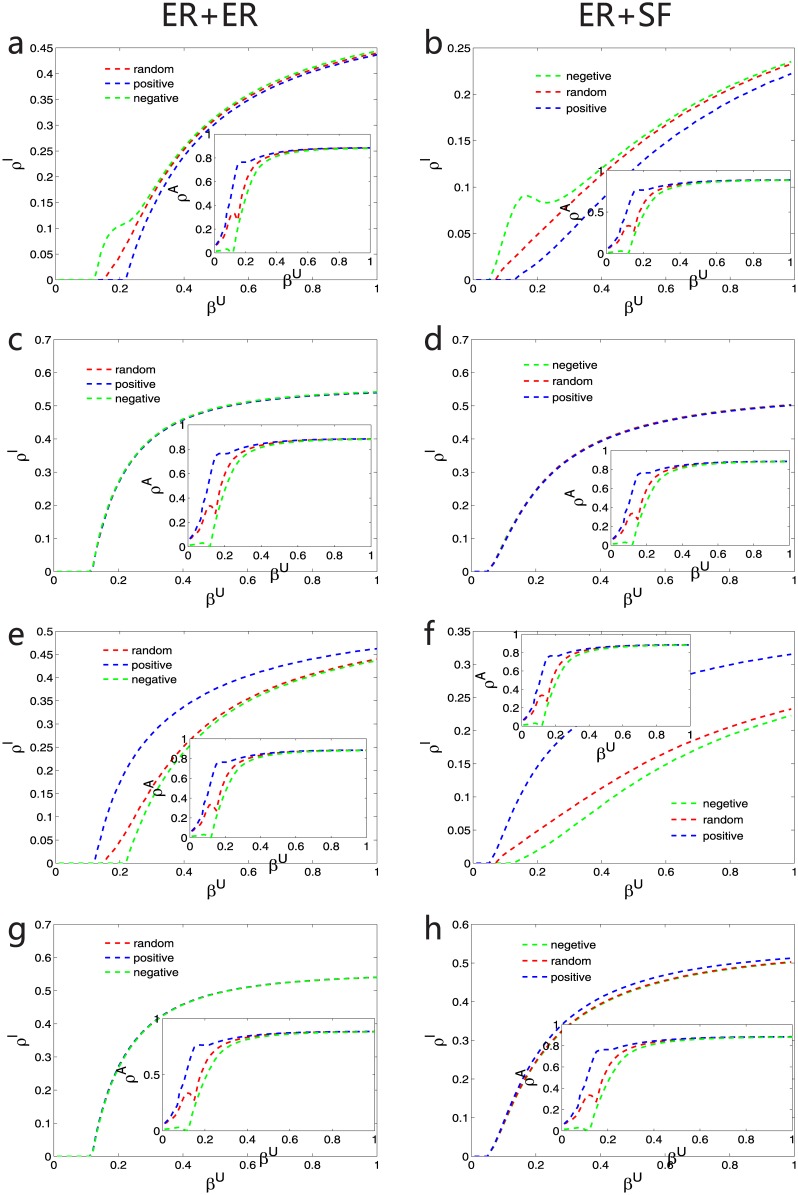
The effects of three heterogeneity models on the coupled dynamic process. Two multiplex networks with 10^4^ nodes on each layer were generated, one with an ER layer+ER layer structure and one with an ER layer+SF layer structure. The results for the four cases of different combinations of heterogeneity measures are shown as follows for each multiplex network and for the three different heterogeneity models, namely, a positive relationship (blue), a negative relationship (green) and a random relationship (red): (a) (b) degree versus degree, (c) (d) degree versus k-core, (e) (f) k-core versus degree, and (g) (h) k-core versus k-core. Each line represents the average of 50 independent Monte Carlo simulations.

To ensure that we obtained uncorrelated multiplex networks, we generated different types of networks to use as the two layers, including SF networks and ER networks [50]. Through many simulations of these multiplex networks, we obtained consistent results regarding the spreading process. As shown in Fig 5, when the k-core measure is used on the contagion layer, the epidemic spreading process is robust to the heterogeneity model and the final epidemic size is larger. In addition, the awareness spreading process reaches a steady state as *β* becomes sufficiently large, for example, *β* = 0.8. Although various studies have shown that interlayer correlations can have a large impact on multiplex networks, these findings demonstrate that the robust spreading process is also ‘robust’ to interlayer correlations.

**Fig 6:**
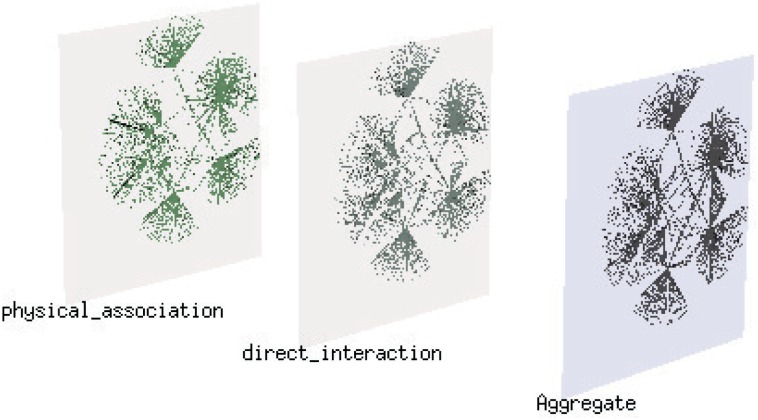
The HIV1 multiplex network. From left to right, the layers are the physical_association layer, the direct_interaction layer and the aggregated network of the two layers, respectively. We treat the physical_association layer as the contagion layer and the direct_interaction layer as the awareness layer.

Below, to illustrate the different effects of the k-core measure and the degree measure, we consider a human HIV1 multiplex network as a realistic example [48, 49]. The multiplex network has five layers, each representing an interaction between genetic and protein-related factors in humans; to simulate our coupled dynamic process, we chose only two layers of this network, as shown in Fig 6. Because this multiplex network is neither a maximally positively correlated network nor an uncorrelated network, exploring it can help us to gain a more comprehensive understanding of the effects of the different measures.

**Fig 7:**
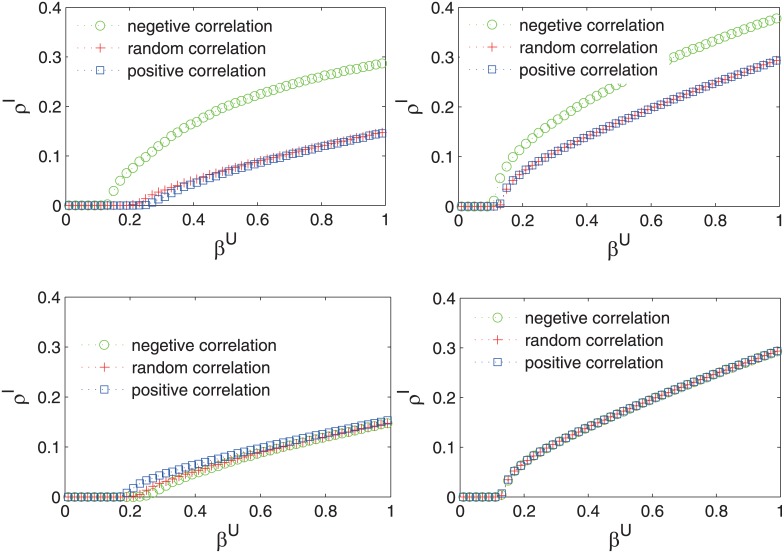
Effects of different heterogeneity measures on the coupled dynamic process. The percentage of infected nodes *ρ*^*I*^ as a function of the infectivity *β* in the following four different cases: (a) degree versus degree, (b) degree versus k-core, (c) k-core versus degree, and (d) k-core versus k-core. In each panel, the green circle-dotted line, the red cross-dotted line and the blue square-dotted line represent the negative correlation model, the random correlation model and the positive correlation model, respectively.

Because in the heterogeneous LACS model, the interactions between nodes on the two layers are one-to-one in nature, we made some changes to the original multiplex network (for more details regarding the data, see the Methods section). Then, we simulated the spreading process on the multiplex network under the four cases of different combinations of heterogeneity measures. Consistent with the results for the synthetic networks, the results for the HIV1 multiplex network, which are shown in Fig 7, present empirical evidence that when the k-core measure is used on both layers, the spreading process is robust to different models. At the same time, the final percentage of infected nodes is larger when the k-core measure is used on the contagion layer, as also observed for the synthetic networks.

## Discussion

In summary, in this paper, we explored the effects of node heterogeneity on epidemic spreading considering an awareness cascade in a multiplex network. Using the k-core measure and the degree measure, we classified the nodes into different groups. Members in the same group were assigned the same infectivity or local awareness threshold values. To gain a comprehensive understanding of the coupled dynamic process, three models were proposed to determine the local awareness thresholds, namely, a linear positive correlation model, a linear negative correlation model and a random correlation model. We found that when the k-core measure is used on the contagion layer, the spreading process is ‘robust’ to different models and the final number of infected nodes is larger, whereas when the degree measure is used on the contagion layer, the use of different models results in significantly different affects on the spreading process. In particular, when the degree measure is used on both the contagion layer and the awareness layer, epidemic outbreaks occur more quickly in the negative model than in the other models. By contrast, when the degree measure is used on the contagion layer and the k-core measure is used on the awareness layer, outbreaks occur most rapidly in the positive model. Furthermore, these results were crosschecked using correlated and uncorrelated multiplex networks. The findings indicate the importance of accounting for the heterogeneity of nodes when studying a coupled dynamic process. Moreover, using the MMCA approach, the calculation of the epidemic thresholds can be converted into an eigenvalue problem. The results show that this method demonstrates good performance in predicting the trends of the epidemic thresholds and that the accuracy is especially high when the k-core measure is used on the contagion layer.

Because of the ‘robustness’ of the k-core measure on the contagion layer, our findings obtained based on multiplex networks also help us to gain a better understanding of the effects of k-core nodes. The results show that when the k-core measure is used on both layers, the heterogeneity of the awareness thresholds has little effect on the epidemic spreading process. Moreover, because of the coupled information-disease spreading process, when we use the k-core measure, nodes on the contagion layer with higher k-core indexes have a larger probability of becoming infected, whereas this is not always true for nodes on the awareness layer. These findings indicate that when studying the spread of epidemics, because of the robustness phenomenon, different epidemic control strategies should be applied to cope with different network structures.

## Methods

### The MMCA method

**Fig 8:**
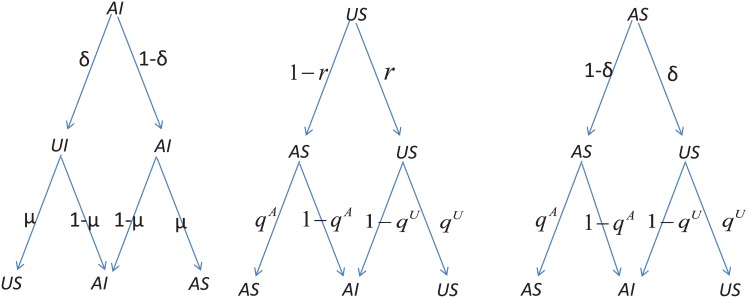
Transition probability trees for the three possible node states. The possible node states are AI (aware and infected), US (unaware and susceptible), and AS (aware and susceptible). Note that *μ* represents the probability of the transition from infected to susceptible, *δ* represents the probability of the transition from aware to unaware, *q*^*A*^ represents the probability that a node will not be infected by its neighbors when it is aware, *q*^*U*^ represents the probability that a node will not be infected by its neighbors if it is unaware, and *r* represents the transition probability for an individual changing from unaware to aware.

In Fig 8, we use a probability tree to illustrate the MMCA method [31]. Because there are only three possible states for every node, namely, US, AI and AS, the probabilities for these states are represented by *p*^*US*^, *p*^*AI*^, and *p*^*AS*^, respectively. According to the probability tree, the evolution equations for these three states for node *i* can be described as follows:

$$\begin{array}{l} p_{i}^{US}(t+1)=p_{i}^{AI}(t)\delta \mu +p_{i}^{US}(t)r_{i}(t)q_{i}^{U}(t)+p_{i}^{AS}\delta q_{i}^{U}(t)\\ p_{i}^{AS}(t+1)=p_{i}^{AS}(t)\mu (1-\delta )+p_{i}^{SU}\left[1-r_{i}(t)\right]q_{i}^{A}(t)+p_{i}^{AS}(1-\delta )q_{i}^{A}(t)\\ p_{i}^{AI}(t+1)=p_{i}^{AI}(t)(1-\mu )+p_{i}^{AS}(t)\left\{\delta \left[1-q_{i}^{U}(t)\right]+(1-\delta )\left[1-q_{i}^{A}(t)\right]\right\}\\ \;+p_{i}^{US}(t)\left\{\left[1-r_{i}(t)\right]\left[1-q_{i}^{A}(t)\right]+r_{i}(t)\left[1-q_{i}^{U}(t)\right]\right\} \end{array}$$ (4)

Therefore, we can obtain the epidemic threshold *β*_*c*_ by letting *t* → ∞ such that the dynamic process reaches a steady state, which means that the coupled equations can be reduced to $p_{i}^{AI}{\left(t+1\right)}_{t\to \infty }=p_{i}^{AI}{\left(t\right)}_{t\to \infty }=p_{i}^{AI}$, $p_{i}^{AS}{\left(t+1\right)}_{t\to \infty }=p_{i}^{AS}{\left(t\right)}_{t\to \infty }=p_{i}^{AS}$, and $p_{i}^{US}{\left(t+1\right)}_{t\to \infty }=p_{i}^{US}{\left(t\right)}_{t\to \infty }=p_{i}^{US}.$ Note that near the epidemic threshold *β*_*c*_, the probability that node *i* will become infected is $p_{i}^{AI}=\epsilon_{i}\ll 1$. In addition, we obtain the probabilities $q_{i}^{A}$ and $q_{i}^{U}$ that node *i* will not be infected, which are described by Eqs (2) and (3), respectively, in accordance with the assumptions above as follows:

$$\begin{array}{c} q_{i}^{A}=\Pi_{t\to \infty }\left(1-b_{ji}p_{j}^{AI}\left(t\right)\beta_{i}^{A}\right)\approx \left(1-\beta_{i}^{A}\sum_{j}b_{ji}\epsilon_{j}\right)\\ q_{i}^{U}=\Pi_{t\to \infty }\left(1-b_{ji}p_{j}^{AI}\left(t\right)\beta_{i}^{U}\right)\approx \left(1-\beta_{i}^{U}\sum_{j}b_{ji}\epsilon_{j}\right) \end{array}$$ (5)

Hence, with respect to Eqs (4) and (5), the stationary probabilities $p_{i}^{AS}$ and $p_{i}^{US}$ are given by

$$\begin{array}{l} p_{i}^{US}=p_{i}^{US}r_{i}+p_{i}^{AS}\delta \\ p_{i}^{AS}=p_{i}^{US}(1-r_{i})+p_{i}^{AS}(1-\delta )\\ \mu \epsilon_{i}=(p_{i}^{AS}\beta^{A}+p_{i}^{US}\beta^{U})\sum_{j}b_{ji}\epsilon_{j} \end{array}$$ (6)

Because $p_{i}^{AI}+p_{i}^{AS}+p_{i}^{US}=1$, where $p_{i}^{AI}+p_{i}^{AS}=p_{i}^{A}$, we find that $p_{i}^{AS}\approx p_{i}^{A}$; then, the probability $p_{i}^{AI}$ can be written as

$$\begin{array}{c} \mu \epsilon_{i}=\beta^{U}\left[1-\left(1-\gamma_{i}\right)p_{i}^{A}\right]\sum_{j}b_{ji}\epsilon_{j} \end{array}$$ (7)

which means that the epidemic threshold $\beta_{c}^{U}$ is written as

$$\begin{array}{c} \beta_{c}^{U}=\frac{\mu }{\Lambda_{max}} \end{array}$$ (8)

where Λ_*max*_ is the maximal eigenvalue of the matrix *S*, whose elements are $s_{ji}=[1-\left(1-\gamma_{i}\right)p_{i}^{A}]b_{ji}$.

### The k-core decomposition

As a result of k-core decomposition, each node is assigned a k-core index value of *k*_*s*_, which represents its location. We can obtain the k-core index through the following steps: Initially, all nodes of degree 1 are assigned the same k-core value, *k*_*s*_ = 1. After successive pruning of these nodes, some nodes with only one link may exist, and upon iterating the process until the degree of each node is greater than 1, these removed nodes are all assigned to a k-core with an index of *k*_*s*_ = 1. In a similar way, we can find the other k-cores with higher indexes *k*_*s*_ by iteratively pruning all nodes in the network with *k* ≤ *k*_*s*_. The process is terminated when all nodes have been pruned.

### The details of the HIV1 multiplex network

The full multiplex network consists of five layers and includes 1005 nodes and 1355 edges in total. In our analysis, we used only two layers, namely, the physical_association layer (contagion layer) and the direct_interaction layer (awareness layer). To ensure one-to-one interactions between the two layers, meaning that the nodes in each of the two layers are the same, we compared the two layers, and upon finding any node that was present in only one layer, we added this node and its connections in that layer to the other layer. After this process, the contagion layer contained 939 edges and the awareness layer contained 1043 edges. Because the awareness layer contained more links than the contagion layer, it was suitable for simulating the spread of information.

## Supporting Information

S1 File S1_File.zip. The data sets of Human HIV1 [48, 49] used in this paper.
(ZIP)
