# Supplementary material for: The Role of Node Heterogeneity in the Coupled Spreading of Epidemics and Awareness
Source: PLoS One. 2016 Aug 12;11(8):e0161037. doi: 10.1371/journal.pone.0161037 (PMC4982672; doi:10.1371/journal.pone.0161037)
Supplement: S1 File — The data sets of Human HIV1 [48, 49] used in this paper. (ZIP) [file pone.0161037.s001.zip › HumanHIV1_Multiplex_Genetic/README.pdf]

HUMAN-HIV1 MULTIPLEX GPI NETWORK

Last update: 1 July 2014

Reference and Acknowledgments

This README file accompanies the dataset representing the multiplex genetic and protein interactions network of the human HIV type 1. If you use this dataset in your work either for analysis or for visualization, you should acknowledge/cite the following papers:

```
“Biogrid: a general repository for interaction datasets”
C. Stark, B.-J. Breitkreutz, T. Reguly, L. Boucher, A. Breitkreutz, and M.
Tyers.
Nucleic Acids Research 2006 34 (1) D535–D539

“MuxViz: A Tool for Multilayer Analysis and Visualization of Networks”
Manlio De Domenico, Mason A. Porter, and Alex Arenas
Journal of Complex Networks 2015 3 (2) 159-176
```

that can be found at the following URLs:

http://nar.oxfordjournals.org/content/34/suppl_1/D535.abstract

http://comnet.oxfordjournals.org/content/3/2/159

This work has been supported by European Commission FET-Proactive project PLEXMATH (Grant No. 317614), the European project devoted to the investigation of multi-level complex systems and has been developed at the Alephsys Lab.

Visit

PLEXMATH: http://www.plexmath.eu/

ALEPHSYS: http://deim.urv.cat/~alephsys/

for further details.

Description of the dataset

We consider different types of genetic interactions for organisms in the Biological General Repository for Interaction Datasets (BioGRID, thebiogrid.org), a public database that archives and disseminates genetic and protein interaction data from humans and model organisms. BioGRID currently includes more than 720,000 interactions that have been curated from both high-throughput data sets and individual focused studies using over 41,000 publications in the primary literature. We use BioGRID 3.2.108 (updated 1 Jan 2014). The present folder concerns human HIV type 4.

The multiplex network used in the paper makes use of the following layers:

1. Physical association
2. Direct interaction
3. Colocalization
4. Association
5. Suppressive genetic interaction defined by inequality

There are 1005 nodes, labelled with integer ID between 1 and 1005, and 1355 connections. The multiplex is directed and unweighted, stored as edges list in the file

```
humanHIV1_genetic_multiplex.edges
```

with format

```
layerID nodeID nodeID weight
```

(Note: weight is 1 for all edges)

The IDs of all layers are stored in

```
humanHIV1_genetic_layers.txt
```

The IDs of nodes, together with their name can be found in the file

```
humanHIV1_genetic_nodes.txt
```

License

This HUMAN-HIV1 MULTIPLEX GPI DATASET is made available under the Open Database License: http://opendatacommons.org/licenses/odbl/1.0/. Any rights in individual contents of the database are licensed under the Database Contents License: http://opendatacommons.org/licenses/dbcl/1.0/

You should find a copy of the above licenses accompanying this dataset. If it is not the case, please contact us (see below).

A friendly summary of this license can be found here:

http://opendatacommons.org/licenses/odbl/summary/

and is reported in the following.

ODC Open Database License (ODbL) Summary

This is a human-readable summary of the ODbL 1.0 license. Please see the disclaimer below.

You are free:

- To Share: To copy, distribute and use the database.
- To Create: To produce works from the database.
- To Adapt: To modify, transform and build upon the database.

As long as you:

- Attribute: You must attribute any public use of the database, or works produced from the database, in the manner specified in the ODbL. For any use or redistribution of the database, or works produced from it, you must make clear to others the license of the database and keep intact any notices on the original database.
- Share-Alike: If you publicly use any adapted version of this database, or works produced from an adapted database, you must also offer that adapted database under the ODbL.
- Keep open: If you redistribute the database, or an adapted version of it, then you may use technological measures that restrict the work (such as DRM) as long as you also redistribute a version without such measures.

Contacts

If you find any error in the dataset or you have questions, please contact


```
Manlio De Domenico
Universitat Rovira i Virgili
Tarragona (Spain)
```

email: manlio.dedomenico@urv.cat

web: http://deim.urv.cat/~manlio.dedomenico/
